# Gun-Shot Wounds of the Jaw and Facial Injuries

**Published:** 1920-04-17

**Authors:** 


### GUN-SHOT WOUNDS OF THE JAW AND FACIAL INJURIES.

### III. Some Nursing Aspects.

### Dietetic Treatment.

A proper dietary for a number of patients suffering in varying degrees from this type of injury constitutes a problem requiring a great amount of careful study. It is a subject interesting, and at the same time most difficult; the control, allocation and avoidance of waste forms not the least arduous task of the sister-in-charge. In the early stages the dietary combines with the medical treatment in increasing the powers of resistance against the onset of latent complications and in restoring the debilitated physical condition of the patient. He is kept in bed and systematically nourished and physicked. Later the dietary will co-operate with the dental treatment in the process of re-educating the jaw and facial muscles in their normal movements in conjunction with unaccustomed mechanical appliances in the mouth, with the least amount of strain upon the newly joined fractures. Thus the dietary assumes a position of importance as an auxiliary to treatment.

Patients are admitted very ill, in a condition of mental and physical collapse, unfit for treatment until they have been sufficiently nourished. Of their own effort they are absolutely helpless. A considerable amount of facial substance may have been shot away, the power of mastication, of swallowing, destroyed, the saliva pouring away, and the patient bordering upon starvation. Therefore he must be fed, and of necessity by artificial methods, the œsophageal or nasal tube, according to the extent of his injury and the minimum of disturbance to him. A preliminary wash out to the mouth and nose is given, and some difficulty must be anticipated in the first introduction of the feeding tube. In his highly nervous state the patient will be fearful of additional hurt and pain, and may resist; but the competence of the nurse, with firm, gentle persuasion, must overcome his fears. Confidence is so quickly gained that in a very short time the introduction of the tube is performed by the patient himself. Every patient artificially fed should have a separate tube reserved for his use alone, which before and after each meal is cleaned and sterilised. An ordinary hospital feeding cup substituted for the glass funnel makes the apparatus appear less formidable to the patient, and is not so easily broken. Indigestion is nearly always more or less present, and some cases have to be studied with particular care with regard to the preparation and suitability of their food. A mixture of rhubarb, soda, and gentian, with a minim of creosote, will be found very useful; and it also must be remembered that the stomach, as well as the mouth, needs to be kept aseptic by a simple treatment of mild purgatives. Associated with the dietary is the question of weight, the loss or gain of which is not wholly related to quantity and quality of food. Although restricted to a menu entirely composed of fluids, it is possible for a steady and gradual increase of weight to occur when the general health conditions are once established. The general dietary must be nourishing and sufficient, easily digested, with plenty of variety; peptonisation is not considered generally necessary, except for cases in which it is specially indicated. The principal basis of the dietary consists of milk, eggs, strong soups, and sugar for those in the early stages, and these can be converted or modified into Benger's Food, strained arrowroot, cornflour containing beaten eggs or stimulants—brandy or port wine (as ordered), bovril made with milk, etc. As the treatment advances the dietary will become semi-solid to solid, but it is by no means a straightforward process; patients frequently have to revert from solids to fluids, due less to mechanical disabilities than to their weakened digestive powers.

An excellent system has been adopted in which the patients are divided into four groups, respectively A, B, C, D, for dietary purposes.

A. Diet composed entirely of fluids.

B. Modified fluids, with very finely minced meat.

C. Well-stewed meats, chicken or rabbit, with well-mashed vegetables; light suet puddings, also toast. At this stage the patient is advanced sufficiently to educate the masticatory powers. Toast is very useful in teaching the patient to bite.

D. Ordinary diet, modified to the powers of the patient.

It is to be noted that patients should not be discharged from hospital as cured until they can with ease assimilate an ordinary diet.

The following is an illustration of the dietary in use at the Croydon Hospital for facial injuries :—

| | Breakfast | Dinner | Tea | Supper |
|---|---|---|---|---|
| Sunday ... | Egg and milk | Chicken broth<br>Blanc mange<br>Fruit | Rice milk | Arrowroot milk |
| Monday ... | Milk gruel | Beef tea<br>Junket | Crumbled bread and milk | Benger's |
| Tuesday ... | Crumbled bread and milk | Rice soup<br>Custard | Sago milk | Cocoa jelly |
| Wednesday | Arrowroot milk | Savoury Custard | Wheatmeal flour gruel | Bread and milk |
| Thursday... | Porridge and milk or syrup and sugar | Chicken broth<br>Apple custard | Rice milk | Coffee jelly |
| Friday ...... | Egg and milk | Beef tea<br>Oatmeal | Bread and milk | Arrowroot and milk |
| Saturday . | Bread and milk | Chicken broth<br>Arrowroot Souffle | Benger's | Egg jelly &c. |

### Cosmetics and Conclusion.

When sufficiently restored in bodily health patients should be encouraged to be up and about taking part in the normal life around them. Provision should also be made for them to follow some mild avocation or interesting hobbies which will help in keeping the mind active and alert, and prevent the self-centredness to which they are prone. Progress and treatment are very slow, discouraging to the patient when extending over a lengthy period of time; not infrequently he has to go back for a time rather than forward. Diversion and occupation therefore become necessary to mental stimulation. Every man can be taught the hygiene and proper care of his mouth, and later be made responsible for the cleansing and adjustment of his oral appliances under expert supervision. Virtually he becomes a daily out-patient, with periods of confinement to hospital when undergoing further prosthetic or plastic treatment in the final processes of facial restoration.

Splints for the correction of the jaw and facial deformities are made of vulcanite rubber, metal, German silver, brass wire, bands and ligatures; they are of no

**Gunshot Wounds of the Jaw**—(*continued*).

standardised pattern. Unlike those in use for other limbs these splints have to be planned, constructed, and fitted to meet the requirements of each individual case. They must be devised so as to be simple yet practical, holding the fractured parts firmly, easy to clean, and comfortable to wear. When fixed it is for the nurse to see they are kept in the position required, and at all times clean and wholesome. Throughout every process the occlusion of the teeth and the "bite" is the guide to perfect position, the "bite" being the true test, *i.e.* the teeth (or imaginary) of the upper jaw meeting those of the lower in correct relationship. The importance of following this golden rule cannot be over-emphasised. Failure results in deformities and distortion of the facial contours; it will prevent the normal function of the jaw and tongue, and thus restrict efficient mastication and power of articulation. In conjunction with the interior splinting is the outward cosmetic repair or plastic treatment of suturing the soft parts and filling in gaps; puckered and scarred tissue is dissected and smoothed, the whole process of repairing and fashioning of the features pursued. This procedure may involve the necessity of placing an artificial eye, the reconstruction of a bridge to the nose and also the nose itself, the reformation of the upper lip, chin, and cheeks. In fashioning the features those "aids" already referred to are used to assist the muscular tension, exerting it in the right direction, and relieving all strain upon the sutures. A simple and efficient support was made in the following manner. On the edge of strong bandage, or muslin (as used for plaster of paris bandage), are sewed common white dressmaker's hooks close together, two pieces being required about 3 inches in length. Each piece is attached either side of the wound with collodion and the gap between bridged by strong silk ligature laced through the hooks.

Much of the operative work will be performed under local anæsthesia (Novocain solution); the value of such a method is greatly appreciated, as the patient is spared many of the dangers and discomforts of ether or chloroform, and more important still it obviates post-operative sickness. Sickness after the suturing of delicate parts of soft tissue is to be avoided as being fatal to good union and healing by first intention.

Fitting the mouth with permanent dentures and artificial teeth is followed by adapting the patient to the use of these permanent appliances necessary to mastication and also the clear articulation of syllables in the formation of words and sentences. Success in regaining the command of speech is a matter of time and great perseverance, entirely due to the individual efforts of the patient.

Connected with the rebuilding and repair of the face, a wide field of study confronts the nurse apart from her ordinary surgical knowledge. Experience, intelligence, and interest are her best guides in following the master mind and gaining a comprehensive knowledge of oral technique, and applying the knowledge so gained to the lasting benefit of men so severely stricken. No type of injury responds so quickly, or compensates the nurse for consistent care. She is kept constantly at work, but her efforts are more than rewarded by the results obtained.

